# Central subfield thickness and cube average thickness as bioimaging biomarkers for ellipsoid zone disruption in diabetic retinopathy

**DOI:** 10.1186/s40942-018-0144-9

**Published:** 2018-11-02

**Authors:** Sukriti Ahuja, Sandeep Saxena, Carsten H. Meyer, Jagjit S. Gilhotra, Levent Akduman

**Affiliations:** 1 Department of Ophthalmology, King George’s Medical University, Lucknow, U.P 226003 India; 2 Department of Ophthalmology, Pallas Klinik, Aarau, Switzerland; 3 Department of Ophthalmology, University of Adelaide, Adelaide, Australia; 4 Department of Ophthalmology, Saint Louis University, St. Louis, USA

**Keywords:** Central subfield thickness, Cube average thickness, Ellipsoid zone, Diabetic retinopathy, Spectral domain optical coherence tomography, Biomarker, Receiver operator characteristic curve, Area under curve

## Abstract

**Background:**

To evaluate the association of central subfield thickness (CST) and cube average thickness (CAT) with ellipsoid zone (EZ) disruption on spectral domain optical coherence tomography (SD-OCT) in patients of diabetic retinopathy (DR).

**Methods:**

Cross sectional study including consecutive patients of type 2 diabetes mellitus [without DR (No DR, n = 97); non-proliferative DR (NPDR, n = 91); proliferative DR (PDR, n = 83)] and healthy controls (n = 82) was undertaken. CST and CAT values were measured using SD-OCT. Data was analyzed using Chi square test, ANOVA and multivariate analysis. Discriminant values of CST and CAT for EZ disruption were evaluated using receiver operator characteristic curve. Area under curve (AUC) was computed.

**Results:**

Mean CAT and CST values in the study subjects showed an incremental trend. Multivariate ordinal logistic regression analysis showed increase in CST (OR = 1.022, p < 0.001) and CAT (OR = 1.029, p < 0.001) as significant independent predictors of EZ disruption. Area under curve showed excellent predictive results of CST (AUC = 0. 943 ± 0.021, 95% CI, 0.902–0.984, p < 0.05) and CAT (AUC = 0.959 ± 0.012, 95% CI 0.936–0.982, p < 0.05), as bioimaging biomarkers, for EZ disruption.

**Conclusion:**

Increase in CST and CAT is associated with increased odds of EZ disruption and these macular parameters serve as bioimaging biomarkers for EZ disruption in DR.

## Background

Diabetic retinopathy (DR) is a micro vascular complication of diabetes mellitus. Presently, approximately 90 million people in the world suffer from DR [1]. The prevalence of DR is expected to rise to 592 million by 2035 [2].

Diabetic macular edema (DME) is a complex pathological process caused by multiple factors, including breakdown of the inner and outer blood retinal barriers, oxidative stress and elevated levels of VEGF. Early detection and treatment of DME can prevent visual loss [3].

Spectral domain Optical coherence tomography (SD-OCT) provides high resolution structural images with precise retinal thickness measurements [4]. It is the technique of choice for early detection of macular edema and for follow-up of diabetic maculopathy. The integrity of ellipsoid zone (EZ) has been found to directly correlate with severity of DR and decrease in best corrected visual acuity (BCVA) [5, 6]. The OCT based macular thickness parameters, namely central subfield thickness (CST) and cube average thickness (CAT) have recently been identified, as bioimaging biomarkers for DME [7]. CST is the preferred OCT measurement for the central macula because of its higher reproducibility and correlation with other measurements of the central macula [8].

A tertiary care center-based cross-sectional study was undertaken to evaluate the association of CST and CAT and EZ disruption on SD-OCT.

## Methods

The authors confirm adherence to the tenets of the Declaration of Helsinki. An institutional review board clearance was obtained. A written informed voluntary consent was received from all the study subjects.

Two hundred seventy-one consecutive patients of diabetes mellitus in the age group of 40 to 65 years were included in the study. Sample size was calculated to be 271 using the formula for sample size calculation [9]. Power of the study was 80%. Diabetes was diagnosed according to American Diabetes Association criteria as a fasting plasma glucose level ≥ 126 mg/dL (7.0 mmol/L) or 2-h post prandial glucose level ≥ 200 mg/dL (11.1 mmol/L) during an oral glucose tolerance test [10]. Based on the fundus photography and fluorescein angiography, subjects were divided into three groups according to the early treatment of diabetic retinopathy study (ETDRS) classification [11]: diabetes mellitus patients without retinopathy (No DR, n = 97), with non-proliferative diabetic retinopathy (NPDR, n = 91), and with proliferative diabetic retinopathy (PDR, n = 83). Healthy controls (n = 82) with no diabetes mellitus were also included. The different OCT systems show discrepancies which can be explained by the differences in the retinal segmentation algorithms. Whereas the Spectralis HRA + OCT and Cirrus HD-OCT include the RPE layer in the retinal segmentation, the other instruments do not. The data imply that the different OCT systems cannot be used interchangeably for the measurement of macular thickness [12]. Thus it is important to have a control group for baseline values.

Patients with any other ocular or systemic diseases affecting the retinal vascular pathology, previous intravitreal injection(s) or any ophthalmic surgical or laser interventions, vitreous hemorrhage and tractional retinal detachment, ischemic heart disease, malignancies, inflammatory disorders, or current or planned dialysis were excluded from the study.

Age, gender, blood sugar status (HbA1c, fasting and post prandial blood sugar) of subjects was documented. BCVA was documented on the logMAR scale. All the study subjects underwent detailed fundus evaluation using stereoscopic slit lamp biomicroscopy and indirect ophthalmoscopy. Digital fundus photography and fluorescein angiography were performed using Zeiss fundus camera FF 450 Plus with a pixel width of 0.0054 and an image size of 2588 × 1958 (Carl Zeiss Meditec AG, Jena, Germany). Study subjects underwent macular thickness analysis using the macular cube 512 × 128 feature of SD-OCT (Cirrus high Definition OCT, Carl Zeiss Meditec Inc., CA, U.S.A). Multiple OCT-derived values were generated for each scan with values corresponding to the average thickness of a macular field. The CST corresponds to the 1 mm diameter center of the fovea and is surrounded by concentric bands of 3 and 6 mm. CAT values were also analyzed.

On SD-OCT, subfoveal retinal photoreceptor EZ disruption was graded into two categories; Grade 1: No EZ disruption, and Grade 2: EZ disruption present. Two experienced observers masked to the status of diabetic retinopathy assessed the grades of EZ disruption.

Statistical analysis: data was analyzed using Statistical Package for Social Sciences (SPSS) version 21.0. Data were expressed as mean ± standard error of the mean (SE). Interobserver correlation for EZ disruption was computed using Spearman rank correlation. Cohen’s kappa coefficient for the study was calculated to be 0.695. Study groups were compared by one-way analysis of variance (ANOVA). Chi square test and ANOVA followed by Tukey’s HSD test was used for univariate intergroup comparisons. Independent predictors for EZ disruption was assessed by multivariate ordinal logistic regression analysis. Discriminant values of CAT, CST and BCVA for EZ disruption, in the study subjects, were evaluated using receiver operator characteristic curve (ROC) analysis and predictive accuracy was calculated by area under curve (AUC). An AUC of 0.90–1 was considered as excellent, 0.80–0.90 as good, 0.70–0.80 as fair, 0.60–0.70 as poor and 0.50–0.60 was a failed test [13]. A ‘p’ value less than 0.05 was considered statistically significant.

## Results

Table 1 shows the characteristics of the study groups. According to ETDRS classification, the cases with retinopathy (174) were classified as mild NPDR (n = 34), moderate NPDR (n = 42), severe NPDR (n = 15), early PDR (n = 66) and advanced PDR (n = 17). 126/174 patients showing features of retinopathy had evidence of DME on SD OCT. ANOVA showed no difference in age among the study groups (F = 1.66, P = 0.183). Chi square test showed similar sex proportions among the study groups (χ2 = 2.05, p = 0.562). ANOVA showed significant difference in logMAR BCVA (F = 105.76, p < 0.001), HbA1C (F = 55.85, P < 0.001), serum urea (F = 4.25, P = 0.008) and creatinine (F = 46.37, P = 0.008) among the patients with diabetes mellitus. ANOVA revealed significant difference in CST (F = 37.11, P < 0.001), and CAT (F = 50.69, P < 0.001) in the patients with diabetes mellitus. No significant difference was found between the controls and No DR patients. Chi square test revealed significant increase in grades of EZ disruption with the severity of diabetic retinopathy (χ2 = 60.60, p < 0.001).

**Table 1:** Characteristics of study groups

S. no.	Characteristic	Controls (n = 82)	No DR (n = 97)	NPDR (n = 91)	PDR (n = 83)
1.	Age (years) (Mean ± SD)	60.31 ± 6.37	57.82 ± 6.98	57.09 ± 9.63	62.25 ± 7.94
2.	Gender				
	Male	58	64	62	59
	Female	24	33	29	24
3.	Glycated Hb (%) (Mean + SD)	5.36 ± 0.53	7.8 ± 0.90	8.45 ± 1.42	8.85 ± 0.82
4.	Serum urea mg/dl	33.21 ± 4.19	33.08 ± 10.2	37.89 ± 4.19	39.88 ± 5.36
5.	Serum creatinine mg/dl	0.96 ± 0.74	1.12 ± 0.14	1.10 ± 0.13	1.62 ± 0.33
6.	BCVA (Log Mar)	0.09 ± 0.09	0.34 ± 0.18	0.70 ± 0.35	1.1 + 0.11
7.	CST (μm)	248.5 ± 12.1	251.1 ± 20.7	290.4 ± 100.7	461.7 ± 82.5
8.	CAT (μm)	255.8 ± 5.0	273.6 ± 36.8	301.1 ± 48.0	371.5 ± 28.8

**Fig. 1:**
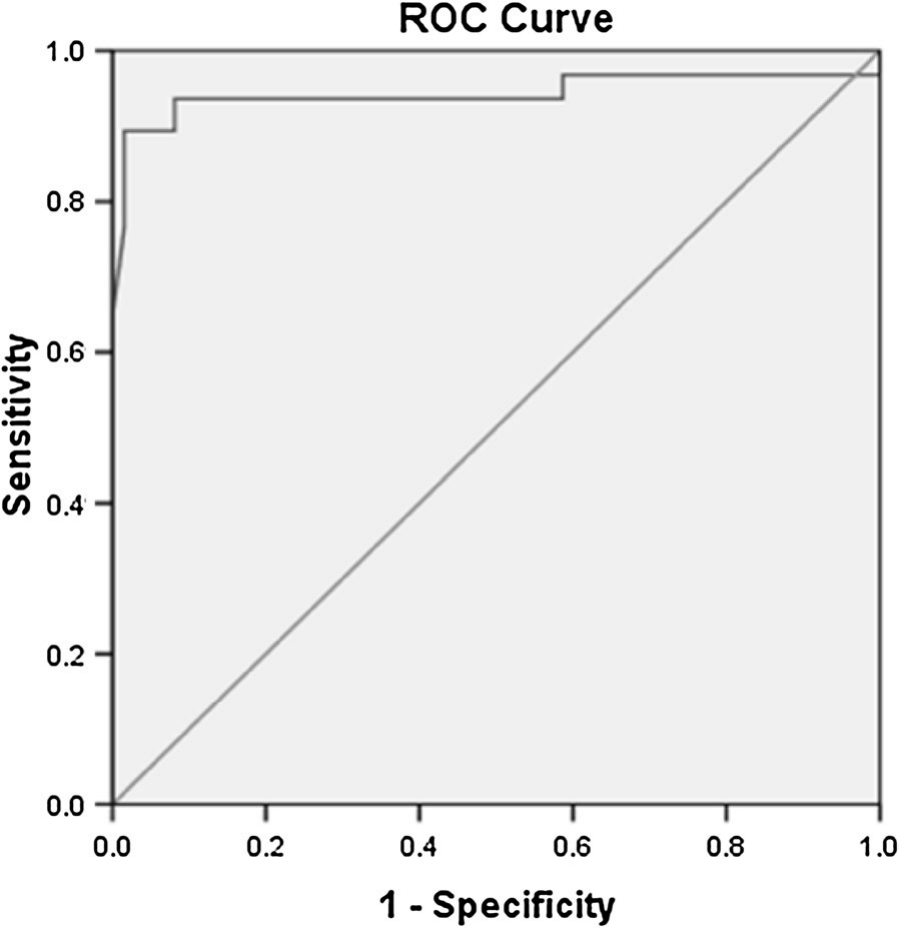
ROC curve showing discriminant value of CST for EZ disruption, AUC = 0. 943 ± 0.021, 95% CI, 0.902–0.984, p < 0.05

**Fig. 2:**
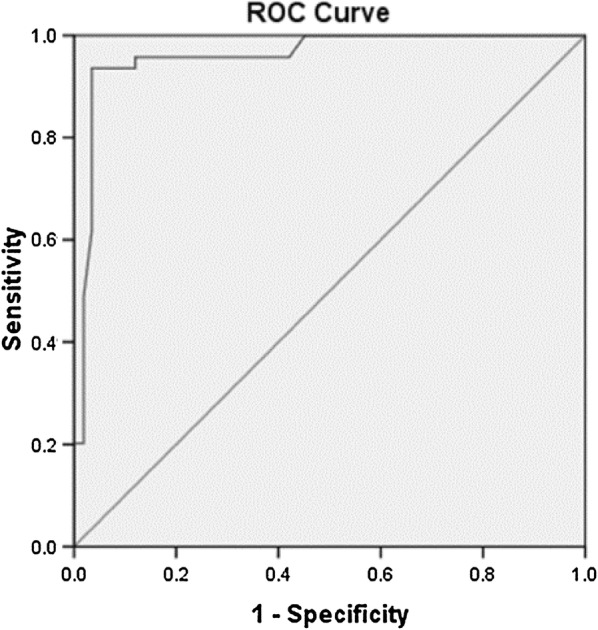
ROC curve showing discriminant value of CAT for EZ disruption, AUC = 0.959 ± 0.012, 95% CI 0.936–0.982, p < 0.05

**Fig. 3:**
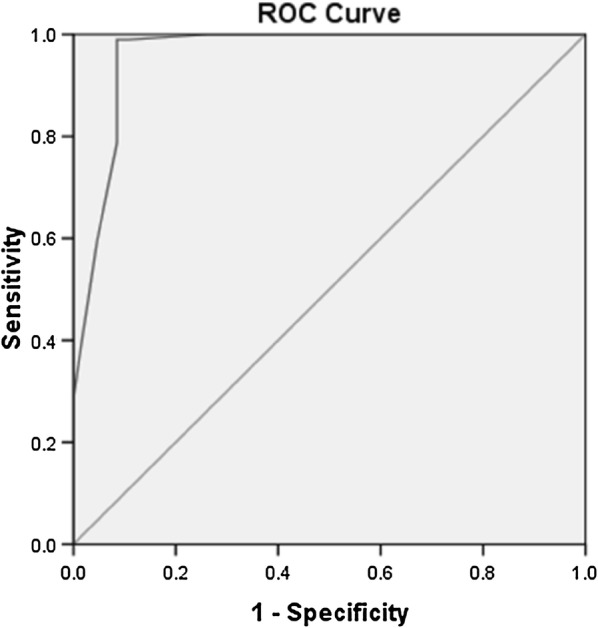
ROC curve showing discriminant value of BCVA for EZ disruption, AUC = 0.961 ± 0.009, 95% CI = 0.943–0.979, P < 0.05

The univariate logistic regression analysis found HbA1c, BCVA, CST, CAT as significant predictors of EZ disruption (p < 0.01). In multivariate analysis, CST (OR = 1.022, P < 0.001) and CAT (OR = 1.029, p < 0.001) showed a significant association with EZ disruption suggesting that CAT and CST serve as significant and independent predictors of EZ disruption. The ROC curve analysis showed diagnostic accuracy of CST [area under curve (AUC) = 0. 943 ± 0.021, 95% CI, 0.902–0.984, p < 0.05] in discriminating subjects with grade 1 and grade 2 EZ disruption (Fig. 1). The ROC curve analysis also revealed significant diagnostic accuracy of CAT (AUC = 0.959 ± 0.012, 95% CI 0.936–0.982, p < 0.05) in discriminating subjects with grade 1 and grade 2 EZ disruption (Fig. 2). For BCVA, AUC for EZ disruption was computed as 0.961 ± 0.009, 95% CI 0.943–0.979, P < 0.05 (Fig. 3).

## Discussion

The study highlighted significant association between the macular thickness parameters (CAT and CST) with EZ disruption in DR, for the first time. CST and CAT increased with increased severity of DR. EZ disruption also increased with increased severity of DR. Intact EZ was observed in all the healthy controls and No DR study groups. EZ disruption was observed in 15/91 (16.4%) patients of NPDR and 79/82 (96.3%) patients of PDR.

Hypoxia, ischemia, accumulation of oxygen free radicals, advanced glycation end products and protein kinase C have been implicated in the pathogenesis of DME [3]. These lead to increased expression of VEGF A resulting in breakdown of blood retinal barrier (BRB) [14]. Breakdown of BRB results in accumulation of plasma proteins which exert a high oncotic pressure in the neural interstitium and produce intraretinal edema. Starling’s law emphasized that the pressure difference between the hydrostatic and oncotic forces of liquid flow is the driving force which contributes to macular edema. When local compensatory mechanisms are overcome, frank vasogenic macular edema develops [3].

VEGF has been considered as the main factor that disrupts the inner blood retinal barrier [15]. Elevated levels of VEGF A correlated with increased vascular permeability, concomitant with decreased zonular occludin-1 levels in vitreous of diabetic patients, which is a main constituent of BRB [16].

In a previous study, it has been demonstrated by OCT Enhanced depth imaging (EDI) that there is a trend of thinning and more irregular retinal pigment epithelium (RPE) and EZ in patients of DME, indicating that along with inner BRB, there is a dysfunction of outer BRB in DME [17]. There was another study demonstrating a microscopic imaging assay for directly visualizing macromolecules leaked through the outer BRB in rodents by microscopic imaging assay. The authors demonstrated the significance of outer BRB breakdown in diabetes [18]. Hyperglycemia induced over activation of Protein Kinase C has also been found to be associated with outer BRB breakdown and photoreceptor degeneration [19].

In the present study, multivariate ordinal logistic regression analysis showed increase in CST and CAT as significant independent predictors of EZ disruption. An increase in CST and CAT was found to be associated with increased odds of EZ disruption. Area under ROC curve showed excellent predictive results of CST and CAT, as biomarkers, for EZ disruption. Disruption of EZ was also found to be associated with significant decrease in BCVA.

An earlier study by Cunha-Vaz et al. [20] observed that the degree of decrease in retinal fluid in the outer layers of retina is a more robust biomarker of BCVA recovery than central retinal thickness, disorganization of inner retinal layer, or EZ disruption. Our earlier studies highlighted that an increase in CST and CAT [6] and EZ disruption, was associated with increased severity of diabetic retinopathy [21–24]. A significant positive correlation was also observed between logMAR visual acuity and grades of EZ disruption [21]. Increased levels of serum VEGF were found to correlate with severity of retinopathy, increased CST and CAT and EZ disruption [21]. In another study, Mori et al. [25] highlighted that intravitreal ranibizumab led to retinal photoreceptor restoration and improvement in BCVA in DME.

## Conclusion

It can be concluded that increase in CST and CAT is associated with increased odds of EZ disruption and these macular thickness parameters serve as bioimaging biomarkers for EZ disruption in diabetic retinopathy. Small sample size was the limitation of the study.

## Abbreviations

VEGF: vascular endothelial growth factor
DR: diabetic retinopathy
DME: diabetic macular edema
NPDR: non-proliferative diabetic retinopathy
PDR: proliferative diabetic retinopathy
CST: central subfield thickness
CAT: cube average thickness
BCVA: best corrected visual acuity
EZ: ellipsoid zone
